# Potential effects of noni (*Morinda citrifolia* L.) fruits extract against obsessive-compulsive disorder in marble burying and nestlet shredding behavior mice models

**DOI:** 10.3389/fphar.2022.993927

**Published:** 2022-09-16

**Authors:** Srikanth Jeyabalan, Logeshwari Bala, Kavimani Subramanian, Sugin Lal Jabaris, Mahendran Sekar, Ling Shing Wong, Vetriselvan Subramaniyan, Kumarappan Chidambaram, Siew Hua Gan, Nur Najihah Izzati Mat Rani, M. Yasmin Begum, Sher Zaman Safi, Siddharthan Selvaraj, Adel Al Fatease, Ali Alamri, Kamini Vijeepallam, Shivkanya Fuloria, Neeraj Kumar Fuloria, Sinouvassane Djearamane

**Affiliations:** ^1^ Department of Pharmacology, Sri Ramachandra Institute of Higher Education and Research (Deemed to be University), Chennai, Tamil Nadu, India; ^2^ Department of Pharmacology, College of Pharmacy, Mother Theresa Post Graduate and Research Institute of Health Sciences, Chennai, Tamil Nadu, India; ^3^ Department of Pharmacology, Siddha Central Research Institute, Central Council for Research in Siddha, Anna Govt. Hospital Campus, Chennai, India; ^4^ Department of Pharmaceutical Chemistry, Faculty of Pharmacy and Health Sciences, Royal College of Medicine Perak, Universiti Kuala Lumpur, Ipoh, Perak, Malaysia; ^5^ Faculty of Health and Life Sciences, INTI International University, Nilai, Malaysia; ^6^ Faculty of Medicine, Bioscience and Nursing, MAHSA University, Jalan SP 2, Bandar Saujana Putra, Jenjarom, Selangor, Malaysia; ^7^ Department of Pharmacology, College of Pharmacy, King Khalid University, Abha, Saudi Arabia; ^8^ School of Pharmacy, Monash University Malaysia, Bandar Sunway, Selangor, Malaysia; ^9^ Faculty of Pharmacy and Health Sciences, Royal College of Medicine Perak, Universiti Kuala Lumpur, Ipoh, Perak, Malaysia; ^10^ Department of Pharmaceutics, College of Pharmacy, King Khalid University, Abha, Saudi Arabia; ^11^ Faculty of Dentistry, AIMST University, Bedong, Kedah, Malaysia; ^12^ Faculty of Pharmacy, AIMST University, Bedong, Kedah, Malaysia; ^13^ Center for Transdisciplinary Research, Department of Pharmacology, Saveetha Institute of Medical and Technical Sciences, Saveetha Dental College and Hospital, Saveetha University, Chennai, Tamil Nadu, India; ^14^ Department of Biomedical Science, Faculty of Science, Universiti Tunku Abdul Rahman, Kampar, Perak, Malaysia

**Keywords:** *Morinda citrifolia*, Noni, neuropharmacology, *Danio rerio*, toxicology, obsessive-compulsive disorder

## Abstract

Obsessive-compulsive disorder (OCD) is a chronic and complex psychiatric disorder that usually includes both obsessions and compulsions. *Morinda citrifolia* L. (Noni) is a functional food and it is a well-known plant due to its potential therapeutic effects on human health in many disorders including neurological and neurodegenerative diseases. The purpose of this study was to evaluate the potential effect of *M. citrifolia* fruits extract (MCFE) against obsessive-compulsive disorder using the marble burying and nestlet shredding behavior mice models. In addition, brain neurotransmitters such as dopamine (DA), serotonin and noradrenaline (NA) were also assessed. Five mice were placed in each of the different groups, and the treatment was given to the animals for a period of 15 days. The marble burying test was evaluated for 30 min on days 1, 7, and 14 while the nestlet shredding test was evaluated for 60 min on days 2, 8, and 15. Treatments with MCFE (100 and 200 mg/kg, p.o.) significantly improved in both behavior tasks when compared to the control group. In addition, diazepam (2 mg/kg, i.p.) and fluoxetine (15 mg/kg, p.o.) were also significantly improved in both tasks when compared with the control mice. Further locomotor activity study revealed that MCFE and fluoxetine did not affect the locomotor functions when compared to vehicle treated mice. In contrast, diazepam significantly decreased locomotion when compared to the control group. The significant amelioration of biogenic amines were observed in the MCFE-treated animals with increased serotonin levels. The histopathology of the brain, liver, and kidney tissues after MCFE administration revealed normal morphological structure with no signs of toxicity or abnormalities. All these results together suggest that MCFE can be a potential drug candidate for the treatment of OCD. Future research should focus on theidentification and the anti-compulsive activity of the constituents from M*. citrifolia*.

## 1 Introduction

Obsessive-compulsive disorder (OCD) is a severe neuropsychiatric disorder defined by unwanted obsessive thoughts and activities. It affects approximately 2–3% of the general population in all civilizations throughout the world (Zheng et al., 2020). Due to its severity, as illustrated by typical repeated behaviors and intrusive thoughts, OCD is an ideal candidate for neuropsychological modelling. Such theories attempt to explain how the symptoms are linked to the underlying information processing impairments. Similarly, the appearance of OCD in head injury setting, localized lesions to the basal ganglia and autoimmune neuropsychiatric diseases raise the possibility of an underlying neurological reason for its genesis and persistence. OCDs that recur frequently may fail to be controlled due to the daily intrusive thought or repeated behavior pattern that most of us can easily dismiss and these disorders have information processing analogues (e.g., in perseveration and inhibition). Modeling them in neuropsychological terms may enable a better understanding to manage OCD (Bottemanne and Arnould, 2021). Although anomalies in the corticostriatal neurocircuitry and serotonin neurotransmission define OCD, abnormalities in the frontal-limbic neurocircuitry are more commonly associated with anxiety. OCD is also linked to a specific pattern of special functioning impairments, such as decreased mental flexibility, reactive inhibition deficiencies as well as planning abilities. To date, drugs acting on the serotonin pathway such as clomipramine and selective serotonin reuptake inhibitors (SSRIs), as well as cognitive-behavioral therapy with exposure and response prevention, are preferred treatments for OCD. On the other hand, other anxiety disorders, respond to a considerably broader spectrum of treatments, including benzodiazepines and psychotherapies other than cognitive behavioral therapies (Goodwin, 2022).

**FIGURE 1:**
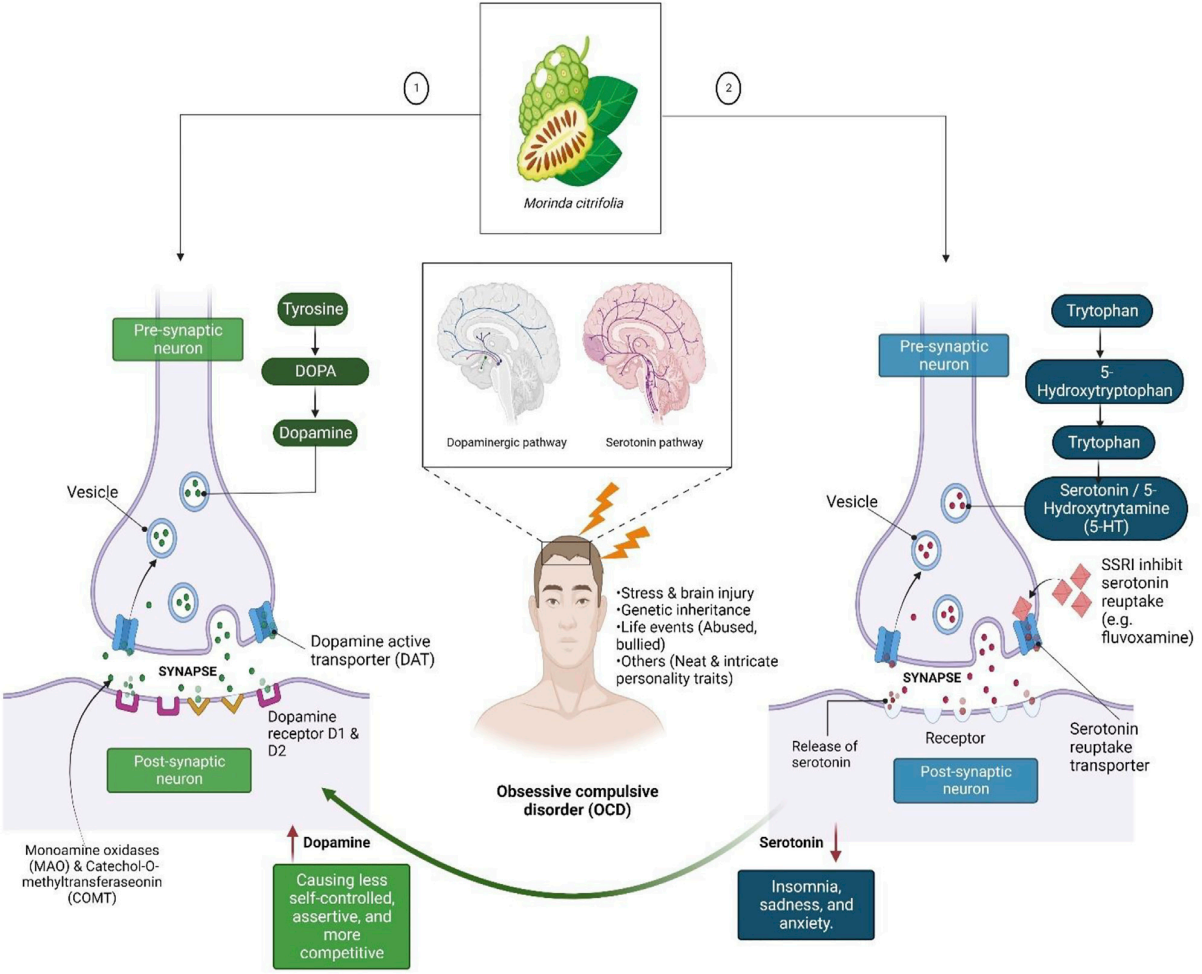
Dopamine (DA) and serotonin neurotransmitters, which are linked to feelings of happiness, motivation, and rewards. DA binds with the D1R and D2R DA receptors after being released into the synaptic cleft by dopaminergic neurons to create excitatory or inhibitory transmission. A DA transporter can influence the amount of DA present, while catechol-O-methyltransferase and monoamine may degrade the DA in the synaptic cleft. Low levels of DA may be linked to poor self-control and other productivity-related issues, whereas low levels of serotonin may cause mood crashes that result in insomnia, melancholy, and anxiety.

Research into the functioning of multiple serotoninergic, dopaminergic and noradrenergic pathways, as well as the functional architecture and structure of corticostriatal pathways across diverse diseases, may lead to a better understanding of OCD. The neurotransmitter serotonin (5-hydroxytryptophan [5-HT]) appears to have a role in the development of OCD (Figure 1). The neurotransmitter 5-hydroxytryptophan (5-HT) or serotonin appears to have a role in the development of OCD. Excessive baseline activity of the excitatory glutamatergic neurons of the orbitofrontal cortex is thought to be present in OCD patients, among other abnormalities. Based on current findings, OCD has malfunctioning 5-HT systems. Thus, SSRIs therapies (e.g., fluvoxamine) reduce OCD symptoms but not drugs that predominantly block dopamine (DA) or noradrenaline (NA) reuptake. Nevertheless, since most SSRIs are only partially effective in treating OCD, it is possible that its major effect is due to regulation of other neurotransmitter systems (Shahini et al., 2021).

DA has emerged as a contender based on its correlation with the serotonin neurotransmitter systems. OCD symptoms are typically present in neuropsychiatric diseases involving DA-rich areas of the basal ganglia and their frontal connections, which led to the discovery of DA as a possible cause of OCD. Several lines of evidence have substantiated its participation. In fact, known connections between the DA and serotonin systems suggest that both neurotransmitter systems are involved in the said disease and its therapy. Nevertheless, the role of dopaminergic neurotransmitter systems in understanding the neuropsychological profile of OCD disorders, particularly the results of visuo-spatial working memory problems, may have direct ramifications (Karpinski et al., 2017).

Medicinal plants have historically played important and sustaining roles in human health, notably in terms of providing both food and therapeutic substances. In actuality, they continue to be the main source of commercial drugs and drug leads (Mushtaq et al., 2018). *Morinda citrifolia* L. (*M. citrifolia* or Noni) is a member of the Rubiaceae family and is found in the Sub-Himalayan tracts of Darjeeling, Konkan and Andaman Islands. In Ayurveda, the Indian mulberry is known as *ashyuka, akshi* and *atchy*, while in Siddha, it is known as *nunaa* or *togaru* (Ali et al., 2013).

The most beneficial product from this plant is purported to be the Noni or Yor juice extract. It is made from fermented *M. citrifolia* and to date, has been reported to have helped more than 10,000 patients recover from a variety of diseases including arthritis, heart disease, diabetes, headaches, muscular pain, high blood pressure, and cancer (Solomon, 1999a; Solomon, 1999b). The study of the Noni improvements in mental-health disorders in animal models has received more attention recently. Studies on humans are relatively rare, and more research, including animal models, is required at the preclinical level. This might be because of its toxic toxicity and poor palatability. Hence, the present study aimed to evaluate the toxicological profile of *M. citrifolia* fruit extract (MCFE) by measuring the levels of the brain neurotransmitters to explore more of its potential anti-OCD-like activity using marble burying and nestlet shredding behavior mice models.

## 2 Materials and methods

### 2.1 Extraction of *M. citrifolia fruits*

The standardized MCFE extract was obtained from Amsar Goa Pvt Ltd. in Goa, India. They declared that Dr. Laxmi Morajkar, Head of the Ayurveda Division, Goa, India, has verified the plant and authenticated with voucher specimen number of AGPL/039/13–14. *M. citrifolia* fruits were stored in an airtight container after being shade-dried in accordance with their procedure. Using a maceration method, extraction was done with water and ethanol in an 80:20 ratio. Briefly, 100 g of shade-dried plant powder and 1 L of hydro alcoholic solvent were combined, and the mixture was placed on a mechanical shaker for 4 h. After that, Whatman No. 1 filter paper was used to filter the solution. The filtrate was then concentrated using flash evaporator and further processed through vacuum desiccators to reach dryness. The yield of MCFE is 4.5 g (4.5%).

### 2.2 I*n vitro* toxicological evaluation

#### 2.2.1 Brine shrimp lethality assay

The lyophilized powder of MCFE (1 g) was dissolved in ethanol (10 ml) and filtered. *Artemia salina* (1 g) cysts were incubated for hatching in a separating funnel filled with sea water. After 24 h, a 0.06% yeast solution was introduced to the hatching chamber to feed the larvae, which was then filled with seawater, followed by a further aeration for 48 h. Subsequently, active nauplii free of egg shells were collected from the hatching chamber and were used in the test.

Using a Pasteur pipette, 10–15 nauplii were extracted from the hatching chamber and were placed on a petri dish containing sea water and a drop of yeast solution. Different concentrations of the plant extract (0.1, 1, 10, 100, and 1,000 μg/ml) and a positive control (potassium dichromate) was made. Then, 0.5 ml of the extract was added to sea water (4.5 ml) and kept at room temperature for 24 h to ensure contact with the active nauplii in the petri dish. Subsequently, the number of surviving nauplii in each petri dish was counted. By comparing the mean surviving larvae of the test and control, the percentage death was obtained. The best-fit line plotting concentration versus percentage lethality provides the 50% lethal concentration (LC_50_) values (Malta et al., 2022).

% Death=[(Number of dead nauplii) / (Number of dead nauplii+Number of live nauplii)]×100

#### 2.2.2 Fish acute toxicity assay

Fish acute toxicity study was carried out as per the OECD test Guideline No. 203. Briefly, adult zebrafish (*D. rerio*) were acclimatized to the laboratory conditions for 7 days under 12–16 h photoperiod and at 21–25°C. The fish were fed twice daily until 24 h before the test started. The amount of dissolved oxygen, pH value and temperature were monitored daily. For the acute toxicity study, ten fish per test concentration (100 mg/L) and control group were maintained. Static renewal method was employed and the test sample was supplied daily to maintain a constant concentration. The fish were inspected after 24, 48, 72, and 96 h. They were deemed as dead should there be no visible movement. Visible abnormalities such as the loss of equilibrium, swimming behavior, respiratory function, pigmentation and other clinical signs were recorded to determine the signs of toxicity during the observation period (Agrawal and Gopal, 2013; Bauer et al., 2021). Finally, toxicity of the entire batch for each concentration was classified by a scale from 0 (no effect), 1 (slight), 2 (medium) and 3 (severe effects).

### 2.3 *In vitro* pharmacological evaluation

#### 2.3.1 Glutamate-induced neurotoxicity assay

Neuroblastoma (SH-SY5Y) cell line was individually plated in Dulbecco’s Modified Eagle Media DMEM media with 1X antibiotic solution and 10% fetal bovine serum in a carbon dioxide (CO_2_) incubator at 37°C with 5% CO_2_. The cells were rinsed in 1X phosphate buffer saline (PBS) (200 μl) followed by a 48 h incubation in various test concentrations of the chemical (100, 200, 400 and 800 μg/ml) in serum-free medium. Prior to glutamate exposure, SH-SY5Y cells were treated with glutamate (20 mM) alone and with various concentrations of test material. At the end of the treatment period, the media was aspirated from the cells. Then, 0.5 mg/ml MTT produced in 1X PBS was added in a CO_2_ incubator followed by incubation at 37°C for 4 h. Subsequently, the MTT-containing media was removed from the cells and rinsed in PBS (200 μl) to produce crystals. The crystals were properly mixed after being dissolved in dimethyl sulfoxide (DMSO, 100 μl). Finally, color intensity was measured at 570 nm where the formazan dye developed a purple blue color using a microplate reader (Magi et al., 2020).

The effect of the samples on the proliferation of various cell lines was expressed as the % cell viability, using the following formula:

% cell viability=(Absorbance of treated cells / Absorbance of control cells)×100

Comparisons were made between the glutamate-induced group with the control and the treatment group of MCFE at 100, 200, 400 and 800 μg/ml. The results were statistically evaluated using one way ANOVA followed by Duncan’s multiple range tests were used to compare treatment and standard groups.

### 2.4 I*n vivo* pharmacological evaluation

Male Swiss albino mice (age 8–12 weeks; 20–25 g) were housed in groups in polypropylene cages (43L x 27 B × 18 H cm) with bedding material and were acclimatized for 5 days prior to the study. The mice were housed in five groups (*n* = 6 for each group) on soft bedding with food and water available *ad libitum*, in a temperature-controlled environment with a light dark cycle of 12:12 h. Following acclimatization, the animals in all groups were continuously treated orally with a standard drug and MCFE for 15 days except for the diazepam group which was treated intraperitonealy (i.p.) only on days 1, 7, and 14. Subsequently, the marble burying test and locomotor activity were measured (Table 1). The nestlet shredding test was performed on days 2, 8, and 15 for all the groups. All experimental procedures were approved by the Institutional Animal Ethical Committee (IAEC), Sri Ramachandra University, constitute as per the directions of the Committee for the Purpose of Control and Supervision of Experiments on Animals (CPCSEA), India, (IAEC NO: IAEC/XLIII/SRU/424/2015). The parameters evaluated for marble burying behavior test was the total number of marbles buried. As for the locomotor activity (actophotometer), two parameters were evaluated 1) total activity score and 2) nestlet shredding test which is the % decrease in weight of the nestlet.

**TABLE 1:** Brine shrimp lethality assay of a standardized MCFE.

Concentration (µg/ml)	% Death of nauplii after 24 h of exposure	
	Potassium dichromate	MCFE
0.10	40.00 ± 1.09	7.42 ± 1.95
1.00	58.33 ± 1.05	14.23 ± 2.02
10.00	67.89 ± 2.93	31.24 ± 2.24
100.00	79.82 ± 1.41	52.62 ± 2.12
1000.00	100.00 ± 1.68	72.34 ± 2.42

Assays were performed in triplicate. Each value represents Mean ± S.E.M.

#### 2.4.1 Marble burying test

The marble burying test takes advantage of the mice’s natural proclivity for digging in the natural settings (e.g., burrows and escape tunnels) and in standard cage bedding. On the other hand, the nestlet shred test takes advantage of the mice’s natural instinct to build nests in order to protect themselves and their offspring from various environmental conditions (e.g. sound, light and temperature). Briefly, male mice were placed in a polypropylene cage (37 cm × 21 cm x 14 cm) with 20 glass marbles (10 mm in diameter) that were evenly dispersed on 5 cm deep sawdust for 20 min without access to food or drink. The testing cages were kept in a separate room from the housing area. The number of marbles buried at least 2/3 of the way within 20 min was used to measure compulsive-like digging behavior. After a 20 min test, the locomotor activity was recorded with an actophotometer for 10 min before the animals were returned to their home cages. The test was conducted on the first, seventh and fourteenth days (Murphy et al., 2017; Wulaer et al., 2018).

#### 2.4.2 Nestlet shredding test

The amount of untorn material for nesting was weighed after male swiss albino mice were given 30 min access to a piece of acotton gauze. Briefly, a single mouse was placed in a cage with a single, pre-weighed nestlet and the cage’s filter-top lid was closed. During the test, the animal was not allowed to eat or drink anything. The mouse was left in the cage with the nestlet for 30 min. Following test completion, the mouse was removed and was restored to its home cage. The remaining intact nestlet material was removed from the cage with forcep and was set aside to dry overnight. To calculate the percentage of nestlet shredded, the remaining unshredded nestlet was weighed and was divided by the initial weight (Foltran et al., 2020).

The % nestled shredding was calculated using the formula:

% Nestlet shredding=[(Initial weight−weight taken after treatment)/ initial weight]×100

### 2.5 *Ex vivo* measurement of brain neurotransmitters

The mice were sacrificed on the day of the experiment where the entire brain was dissected out and the sub-cortical region containing the striatum was separated. A weighed amount of tissue were homogenised for 1 min in hydrochloric acid (HCl)-butanol (5 ml). Subsequently, the sample was centrifuged for 10 min at 2000 rpm. An aliquot of the supernatant phase (1 ml) was then taken and placed in a centrifuge tube with 2.5 ml heptane and 0.3 ml HCl (0.1 M). The tube was centrifuged under similar circumstances as described following a 10 min of vigorous shaking in order to separate the two phases. Then, the overlying organic phase was discarded. Finally, the aqueous phase (0.2 ml) was used for measurement of 5-hydroxytryptamine (5-HT), NA and DA levels.

#### 2.5.1 Measurement of dopamine (DA) and noradrenaline levels

To the 0.2 ml aqueous phase, 0.05 ml HCl (0.4 M) and 0.1 ml ethylene-diaminetetraacetic acid (EDTA)/sodium acetate buffer (pH 6.9) was added, followed by the addition of 0.1 ml iodine solution (0.1 M in ethanol) for oxidation. After 2 min, the reaction was halted by adding 0.1 ml sodium sulfite (Na_2_SO_3_) solution. Then 0.1 ml acetic acid was added after 1.5 min. When the material was returned to room temperature, the excitation and emission spectra were read from the spectrofluorimeter by heating it to 100°C for 6 min. DA was measured at 330–375 nm, while NA was measured at 395–485 nm (Ajibade et al., 2022).

#### 2.5.2 Measurement of serotonin level

To the 0.2 ml aqueous phase, 0.25 ml O-phthalaldehyde (OPT) reagent was applied. The fluorophore was heated to 100°C for 10 min for its development. In the spectrofluorimeter, the readings were taken at 360–470 nm once the samples had reached equilibrium with the ambient temperature. Tissue blanks for DA and NA were made by reversing the sequence of the oxidation reagents (sodium sulphite before iodine). Then, approximately 0.25 ml concentrated HCl without OPT was applied to the tissue blank for serotonin assessments. Three internal standards (500 μg/ml), NA, DA and 5-HT were prepared in distilled water with a 1:2 ratio of HCl-butanol (Bose et al., 2019).

#### 2.5.3 Measurement of total protein

The blue color produced by the reduction of the phosphomolybdic phosphotungstic components in the Folin-Ciocalteu reagent by the amino acids tyrosine and tryptophan in the protein, as well as the color produced by the protein’s biuret reaction with the alkaline cupric tartarate, were measured. Both measurements were read at 660 nm (Deutch, 2018). The total protein concentration was determined using standard kits and Lowry’s Folin-Ciocalteu phenol reagent.

### 2.6 Ex *vivo* measurement of antioxidant enzymes

Using a Potter-Elvehjem homogenizer, the brain, kidney and liver tissues from different treatment groups were homogenised in 10% (w/v) ice-cold cooled sodium phosphate buffer (0.1 M, pH 7.4). A portion of the homogenate was used for biochemical calculations, while the rest was centrifuged at 9,000 rpm for 30 min at 4°C in a Sigma chilled centrifuge to obtain the supernatant, which was then used to calculate lipid peroxide (LPO), superoxide dismutase (SOD), catalase (CAT) and protein levels (Samarghandian et al., 2017).

#### 2.6.1 SOD assay

An ultraviolet (UV) spectrophotometer was used to measure the chromogen’s color intensity at 420 nm. The result was expressed in μmoles/min/mg of protein. Pyrogallol-tris-DETPA, sufficiently diluted tissue and water were included in the assay mixture. The increase in the absorbance at 420 nm was used to calculate the rate of pyrogallol autoxidation. An enzyme unit reduces pyrogallol autoxidation rate by 50% while SOD was measured in units/min/g of protein (Ahmadzadeh et al., 2022).

#### 2.6.2 Measurement of catalase

The tissue was homogenized at 1–4°C with phosphate buffer (15 M) followed by centrifugation. The sediment was agitated with cold phosphate buffer and was left in the cold for several hours, with an occasional shaking. Extraction was performed twice and the supernatants were mixed. In one cuvette, 3 ml of hydrogen peroxide (H_2_O_2_) phosphate buffer was introduced, followed by the addition of 0.01–0.04 ml sample. Subsequently, the results were read at 240 nm against a control cuvette containing enzyme solution without H_2_O_2_ phosphate buffer. The optical density normally dropped (from 0.45 to 0.40) and was recorded (Lairion et al., 2022).

#### 2.6.3 Measurement of lipid peroxide

0.2 ml of tissue homogenate, 1.5 ml of 20% acetic acid, 0.2 ml of 8.1% sodium dodecyl sulphate and 1.5 ml of 8% thiobarbituric acid were added. The mixture was diluted to 4.0 ml with distilled water and then heated in a water bath (95°C) for 60 min. After cooling the tubes to room temperature, the final volume was increased to 5.0 ml in each tube. Then, the n-butanol:pyridine mixture was added and the contents were vortexed for 2 min. The organic upper layer was taken following centrifugation at 3,000 rpm for 10 min and the optical density was measured at 532 nm against the blank. The quantities of lipid peroxides in brain homogenate were measured in nanomoles of malondialdehyde/min/mg protein (Ohkawa et al., 1979).

### 2.7 *Ex vivo* toxicological evaluation

#### 2.7.1 Measurement of serum biochemical and hematological parameters

On the 15th day, mice were anaesthetized using isoflurane, and blood was collected retro-orbitally. Standard kit procedures were used to determine biochemical parameters such as alkaline phosphatase (ALP), alanine aminotransferase (ALT), aspartate aminotransferase (AST), creatinine (CR) and urea. Hemoglobin (Hb), red blood cells (RBC), white blood cells (WBC), mean corpuscular volume (MCV), platelet and hematocrit were measured using standard kit procedures.

#### 2.7.2 Histopathological study

On the 15th day, the animals were euthanized by overdose of isoflurane and the essential organs, including the liver, kidneys, brains, heart, spleen, and lungs, were removed aseptically, cut and washed in ice cold saline. The sections were preserved in a 10% formalin solution. The tissues were cut into 4–6 μm thickness sections and were stained with hematoxylin and eosin after being dehydrated and fixed in paraffin wax. They were photographed and examined under a microscope for histological alterations in liver, brain and kidney architecture.

### 2.8 Statistical analysis

All *in vitro* and *ex vivo* activities were repeated thrice and the results expressed as mean ± S.E.M. The 50% minimum inhibitory concentration (IC_50_) and the LC_50_ were graphically analyzed using a GraphPad Prism (5.0) software. Linear regression was used to determine the dose-response association. The 95% confidence level (*p* < 0.05) was deemed as statistically significant. For *in vivo* trials, the program used a one-way ANOVA followed by Duncan’s multiple range tests to compare between treatment and control groups.

## 3 Results

### 3.1 *In vitro* toxicological evaluation

#### 3.1.1 Brine shrimp lethality assay

Brine shrimp assay was conducted for MCFE while lethality dose was calculated at 24 h by using a standard potassium dichromate solution. The yielded values were entered into GraphPad software. Probit analysis was observed for both potassium dichromate and MCFE (Table 1). The shrimps which were kept in potassium dichromate showed toxic effects against it whereas the shrimps immersed in MCFE did not show much toxicity, as confirmed by the LC_50_ for potassium dichromate and the extract.

The LC_50_ of potassium dichromate and MCFE was 15.69 and 25.29 µg/ml respectively. Based on the correlation findings as reported by Parra et al., for test substances which showed LC_50_ < 10 μg/ml possesses LD_50_ between 100 and 1,000 mg/kg; LC_50_ < 20 μg/ml possesses LD_50_ between 1,000 and 2,500 mg/kg and LC_50_ > 25 μg/ml possesses LD_50_ between 2,500 and 8,000 mg/kg (Ding et al., 2022). The LC_50_ for MCFE was in the range of LC_50_ > 25 μg/ml and therefore the LD_50_ for MCFE was expected between 2,500 and 8,000 mg/kg.

#### 3.1.2 Glutamate-induced neurotoxicity assay

The study demonstrated that MCFE produced neuroprotective activities against the glutamate-induced neurotoxicity in a dose-dependent manner (Table 2). In case of MCFE-treated groups, there was also a dose-dependent increase in percentage cell viability.

**TABLE 2:** Glutamate-induced neurotoxicity assay and neuroprotective effect of MCFE.

Treatment groups	% Cell viability
Control (5% DMSO)	100.00 ± 1.68
Glutamate (20 mM)	42.24 ± 2.38^a*^
Glutamate (20 mM) + MCFE 100 μg/ml	62.34 ± 1.38
Glutamate (20 mM) + MCFE 200 μg/ml	74.32 ± 3.4^b*^
Glutamate (20 mM) + MCFE 400 μg/ml	82.27 ± 2.97^b*^
Glutamate (20 mM) + MCFE 800 μg/ml	91.86 ± 3.24

Values are represented as Mean ± S.E.M of three observations. A one-way ANOVA followed by Duncan’s multiple range tests was used to compare the treatment and control groups. *p*-value^a*^ represents *p* <0.05 compared with the control group. *p*-value^b*^ represents *p* <0.05 compared with the glutamate-treated group.

### 3.2 *In vivo* toxicological evaluation

#### 3.2.1 Fish acute toxicity assay

**FIGURE 2:**
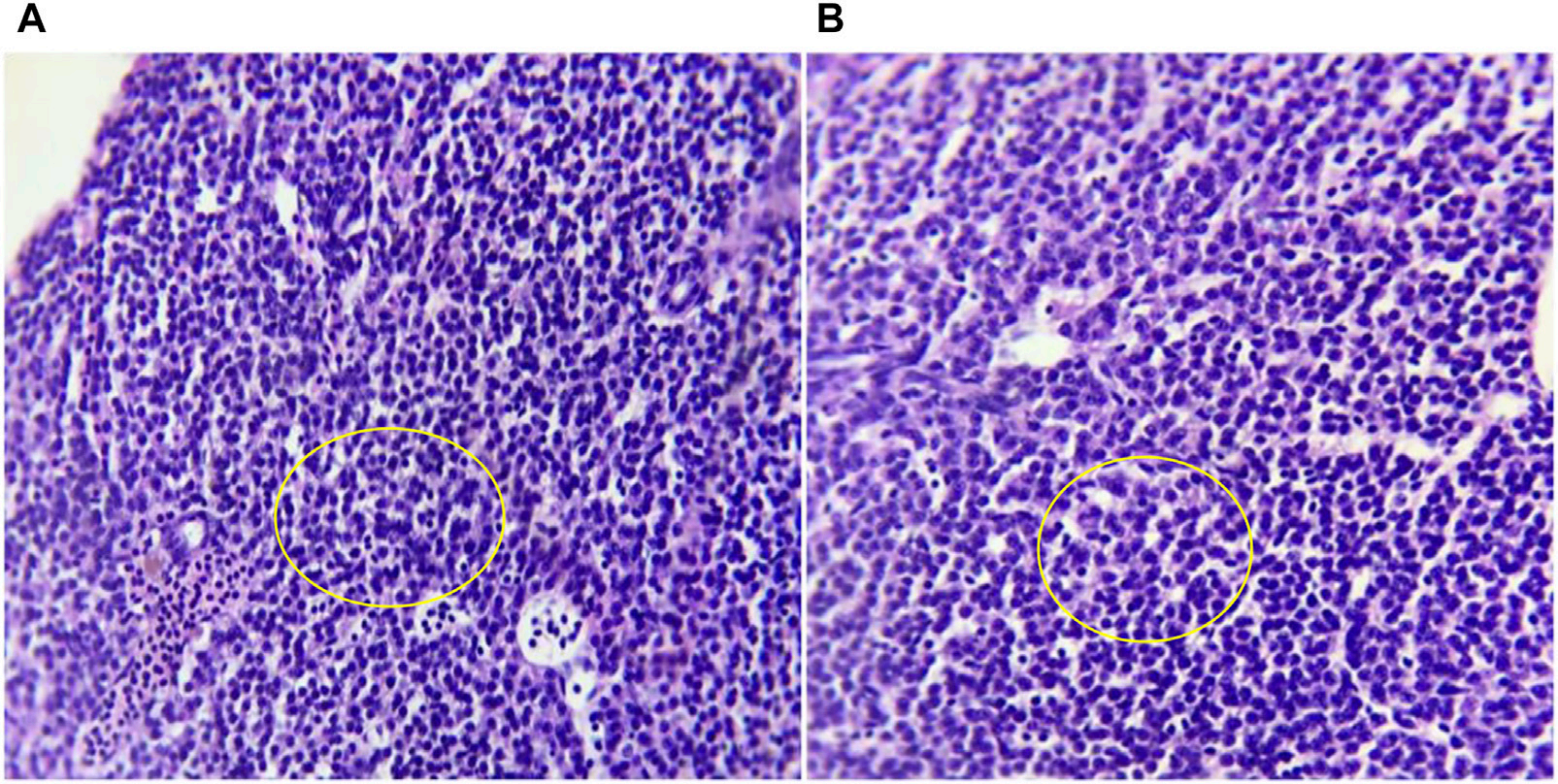
Photomicrographs at 10X magnification of haematoxylin and eosin (H&E) stained Liver histology of **(A)** control and **(B)** MCFE-treated zebra fish. In liver histology both groups shows well-organized polygonal shaped hepatic parenchyma.

As per the OECD test guideline, fish (Test Guideline No. 203) acute toxicity test (limit test) was performed at 100 mg/L of MCFE to demonstrate that the LC_50_ is greater than the said concentration. The visible abnormalities such as loss of equilibrium, swimming behavior, respiratory function, pigmentation, and other clinical signs were observed as per the guideline. No mortality and visible abnormalities were observed in the MCFE-exposed and in control groups during the observation period (i.e., at 24, 48, 72, and 96 h). The liver histology of control and treatment group fish showed normal morphology and organization of hepatocytes. There was no evidence of karyorrhexis and inflammation. Both control and treatment groups have well-organized polygonal shaped hepatic parenchyma [Figures 2A,B].

### 3.3 *In vivo* pharmacological evaluation

#### 3.3.1 Marble burying test

**FIGURE 3:**
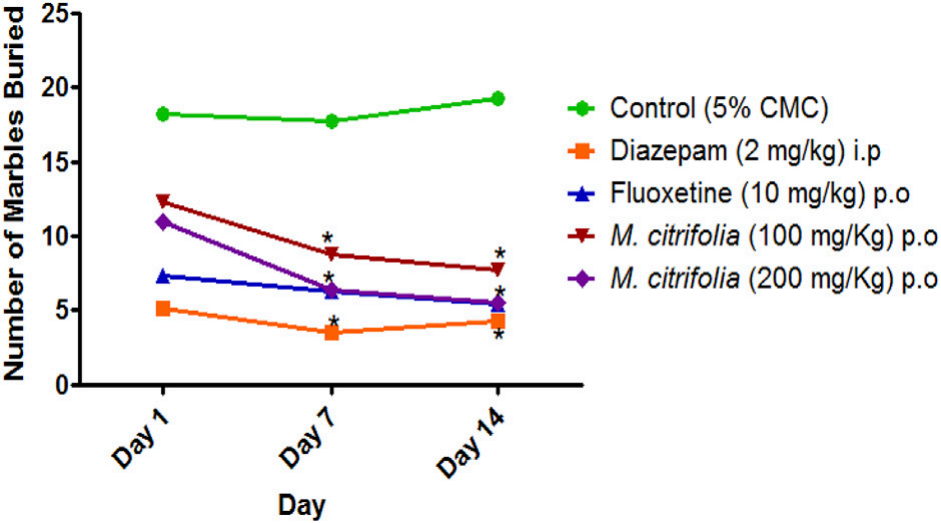
*In vivo* evaluation of marble burying behavior test of MCFE.

**FIGURE 4:**
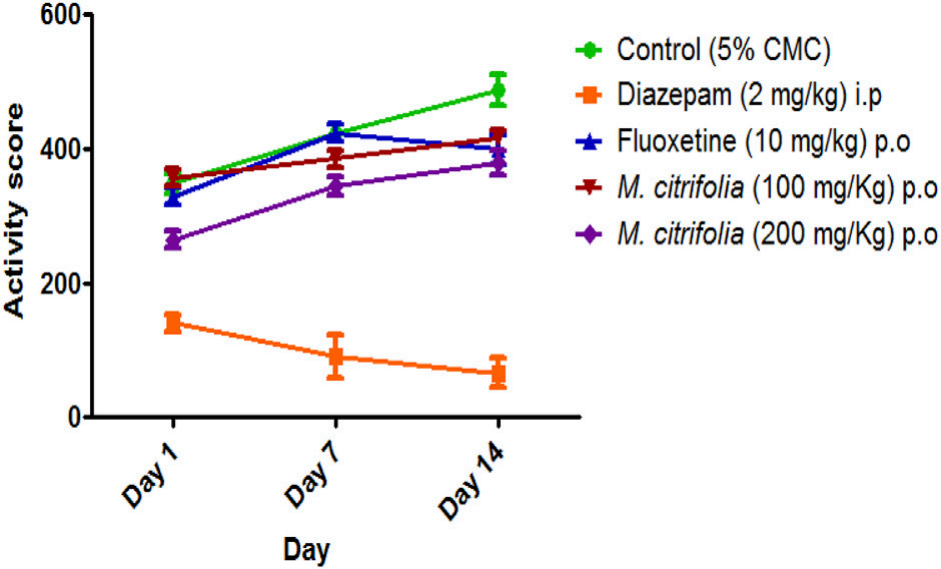
*In vivo* evaluation of locomotor activity of MCFE.

The marble burying test was evaluated for 30 min on 1^st^, 7th and 14th and the total number of marbles buried after a 30 min exposure was observed. There was a significant decrease in the number of marbles buried both in the diazepam-treated (*p* <0.05) and standard fluoxetine-treated (*p* <0.05) groups when compared to the control group (Figure 3). In addition, the MCFE-treated group (100 and 200 mg/kg, p.o.) produced a significant reduction in the number of marbles buried as compared to the standard drug fluoxetine and the negative control group diazepam on the 7th and 14th day. The MCFE-treated group (200 mg/kg, p.o.) has the maximum reduction in the number of marbles buried on day 14. The results of MCFE was depicted in and Figure 4.

#### 3.3.2 Nestlet shredding test

**FIGURE 5:**
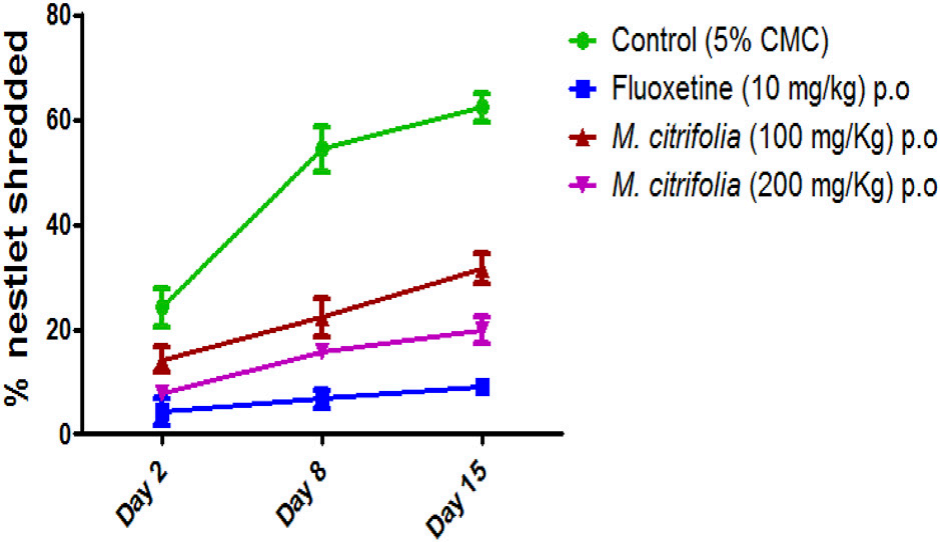
*In vivo* evaluation of nestlet shredding test of MCFE.

The nestlet shredding test was evaluated for 60 min on days 2, 8, and 15. There was a significant decrease in the nestlet shredding in the fluoxetine-treated (*p* <0.05) as compared to the control group. There was a significant difference in the MCFE-treated group (100 and 200 mg/kg, p.o.) as compared to the control group indicating the anti-compulsive effect (Figure 5).

### 3.4 *Ex vivo* measurement of brain neurotransmitters

#### 3.4.1 Measurement of total protein levels

All treatment groups were evaluated for the presence of total protein in the brain (Table 3) which was highest for the fluoxetine group (20.8 ± 1.50).

**TABLE 3:** Measurement of protein, DA, NA and seratonin levels in mice brain on chronic administration of MCFE.

Groups	Protein (mg/dl)	DA (ng/mg protein)	NA (ng/mg protein)	Serotonin (ng/mg protein)
Control (0.5% CMC)	18.6 ± 1.80	32.40 ± 1.40	20.86 ± 1.80	26.50 ± 0.32
Diazepam (2 mg/kg, i.p.)	12.3 ± 2.30*	15.60 ± 1.20*	12.62 ± 2.30*	15.40 ± 2.60*
Fluoxetine (10 mg/kg, p.o.)	20.8 ± 1.50 *	24.64 ± 1.80*	23.82 ± 1.50*	29.60 ± 2.40*
MCFE (100 mg/kg, p.o.)	19.6 ± 1.90*	29.80 ± 1.80*	26.50 ± 1.90*	24.40 ± 2.80*
MCFE (200 mg/kg, p.o.)	18.0 ± 2.10	30.60 ± 2.20*	28.40 ± 2.10*	28.60 ± 1.80*

Values are represented as Mean ± SEM of triplicate samples. A one-way ANOVA followed by Duncan’s multiple range tests was used to compare the treatment and control groups. *p*-value *represents *p* <0.05 compared with the control group (*n* = 6).

#### 3.4.2 Measurement of DA and NA levels

The brain monoamines were measured in the whole brain homogenate of the-treated groups using a spectrofluorimeter. There was a significant decrease in the brain DA and NA levels in the diazepam-treated group as compared to that for control. The fluoxetine and MCFE-treated groups showed a significant decrease in the DA levels while there was a significant increase in NA levels compared to the control groups (Table 3). The increase in NA levels of MCFE-treated groups was in a dose-dependent manner.

#### 3.4.3 Measurement of serotonin levels

Serotonin levels were measured after 15 days treatment. The MCFE-treated group showed a significant increase (*p* <0.05) in the serotonin levels in a dose-dependent manner as compared to the standard drug fluoxetine. The diazepam-treated group showed a significant decrease (*p* <0.05) in the serotonin levels in the brain as compared to the control group. Serotonin levels were higher (28.60 ± 1.80 ng/mg) in animals which received the higher MCFE dose (200 mg/kg, p.o.) (Table 3).

### 3.5 *Ex vivo* measurement of antioxidant enzymes

#### 3.5.1 Superoxide dismutase assay

SOD levels were measured at 420 nm in the brain, liver and kidney tissues of the treated animals after 15 days using a UV spectrophotometer. There was a significant (*p* <0.05) increase in the brain SOD levels for MCFE (100 mg/kg) and 200 mg/kg-treated groups as compared to that of the diazepam-treated group. There was also a significant increase in the liver SOD levels for MCFE (200 mg/kg, p.o.) and the kidney SOD levels as compared to the diazepam-treated groups (Table 4).

**TABLE 4:** Measurement of superoxide dismutase, TBARS and catalase levels in mice brain, liver and kidney on chronic administration of MCFE.

Superoxide dismutase levels			
Group	Brain (U/mg protein)	Liver (U/mg protein)	Kidney (U/mg protein)
Control (0.5% CMC)	11.40 ± 0.32	10.40 ± 0.23	9.20 ± 0.32
Diazepam (2 mg/kg, i.p.)	11.80 ± 2.60	9.80 ± 2.52	10.70 ± 2.60
Fluoxetine (10 mg/kg, p.o.)	14.40 ± 2.40	12.40 ± 2.12	13.50 ± 2.40
MCFE (100 mg/kg, p.o.)	15.60 ± 2.80*	14.60 ± 2.42	13.60 ± 2.80*
MCFE (200 mg/kg, p.o.)	16.70 ± 1.80*	15.70 ± 1.21*	11.40 ± 1.80
**Catalase levels**			
Control (0.5% CMC)	7.28 ± 1.40	8.28 ± 1.32	6.28 ± 1.90
Diazepam (2 mg/kg, i.p.)	8.20 ± 1.20	9.20 ± 1.23	7.20 ± 1.80
Fluoxetine (10 mg/kg, p.o.)	15.60 ± 1.80*	12.60 ± 1.54	14.60 ± 1.20*
MCFE (100 mg/kg, p.o.)	16.40 ± 1.80*	13.40 ± 1.42	18.40 ± 1.80*
MCFE (200 mg/kg, p.o.)	17.80 ± 2.20*	16.80 ± 2.12*	19.80 ± 2.30*
**TBARS levels**			
Control (0.5% CMC)	185.00 ± 8.60	162.00 ± 5.60	175.00 ± 6.60
Diazepam (2 mg/kg, i.p.)	189.60 ± 12.80	171.60 ± 10.80	169.60 ± 11.80
Fluoxetine (10 mg/kg, p.o.)	206.80 ± 9.80*	208.80 ± 8.80*	186.80 ± 7.80
MCFE (100 mg/kg, p.o.)	209.80 ± 3.20	212.80 ± 4.20	199.80 ± 7.20
MCFE (200 mg/kg, p.o.)	212.60 ± 13.60*	216.60 ± 12.60*	192.60 ± 11.60

Values are represented as Mean ± SEM of triplicate samples. A one-way ANOVA followed by Duncan’s multiple range tests was used to compare between the treatment and control groups. *p*-value* represents *p* <0.05 compared with the control group (*n* = 6).

#### 3.5.2 Measurement of lipid peroxidation levels

The thiobarbituric acid reactive substances (TBARS) levels were measured in the brain, liver, and kidney tissues of the treated animal after 15 days using a UV spectrophotometer at 532 nm There was a significant (*p* <0.05) increase in the brain and liver TBARS levels of MCFE-treated groups as compared to that of the diazepam-treated group (Table 4).

#### 3.5.3 Measurement of catalase levels

Catalase levels were measured in the brain, liver and kidney tissues after 15 days by using a UV spectrophotometer at 240 nm. There was a significant increase in the brain catalase levels of fluoxetine, MCFE (100 and 200 mg/kg, p.o.) as compared to that of the diazepam-treated group (Table 4). There was also a significant change in the liver catalase of the MCFE (200 mg/kg, p.o) treated group. A significant change was also observed in the catalase levels of MCFE-treated groups (100 and 200 mg/kg, p.o. in the kidney in a dose-dependent manner.

### 3.6 *Ex vivo* toxicological evaluation

#### 3.6.1 Measurement of serum biochemical and hematological parameters

No significant changes in the biochemical parameters were seen after 15 days treatment with MCFE at both dose levels, indicating the non-toxicological nature of the standardized MCFE (Table 5). There were also no significant changes in the blood parameters evaluated after 15 days treatment with MCFE at both dose levels (Table 6), thus further supporting the non-toxicological nature of the standardized MCFE.

**TABLE 5:** Measurement of biochemical parameters on chronic administration of MCFE.

Group	ALP (U/L)	ALT (U/L)	AST (U/L)	CR (mg/dl)	Urea (mg/dl)
Control (0.5% CMC)	265.09 ± 19.67	81.99 ± 3.16	113.91 ± 6.87	0.86 ± 0.02	31.77 ± 3.10
MCFE (100 mg/kg, p.o.)	269.81 ± 12.89	70.26 ± 0.66	107.23 ± 0.92	0.77 ± 0.07	30.35 ± 2.57
MCFE (200 mg/kg, p.o.)	259.80 ± 8.83	75.28 ± 0.69	110.67 ± 1.92	0.71 ± 0.06	32.68 ± 6.86

Values are represented as Mean ± SEM of triplicate samples. A one-way ANOVA followed by Duncan’s multiple range tests was used to compare the treatment and control groups (*n* = 6).

**TABLE 6:** Measurement of blood parameters on chronic administration of MCFE.

Parameters	Control	MCFE (100 mg/kg) p.o	MCFE (200 mg/kg) p.o
Hb (g/dl)	15.95 ± 0.511	16.50 ± 0.42	13.97 ± 0.30
RBC (10^6^/µl)	8.30 ± 0.1366	9.55 ± 0.23	8.117 ± 0.14
WBC (10^3^/µl)	5477.00 ± 212.9	5667.00 ± 217.10	5800.00 ± 916.20
MCV	43.92 ± 0.14	38.65 ± 0.440	37.53 ± 0.47
Platelet (10^3^/µl)	126.20 ± 3.44	117.50 ± 11.20	136.77 ± 0.23
Hematocrit (%)	37.33 ± 0.66	38.00 ± 0.85	34.00 ± 0.85

Values are represented as Mean ± SEM of triplicate samples. A one-way ANOVA followed by Duncan’s multiple range tests was used to compare the treatment and control groups (*n* = 6).

#### 3.6.2 Histopathological study

**FIGURE 6:**
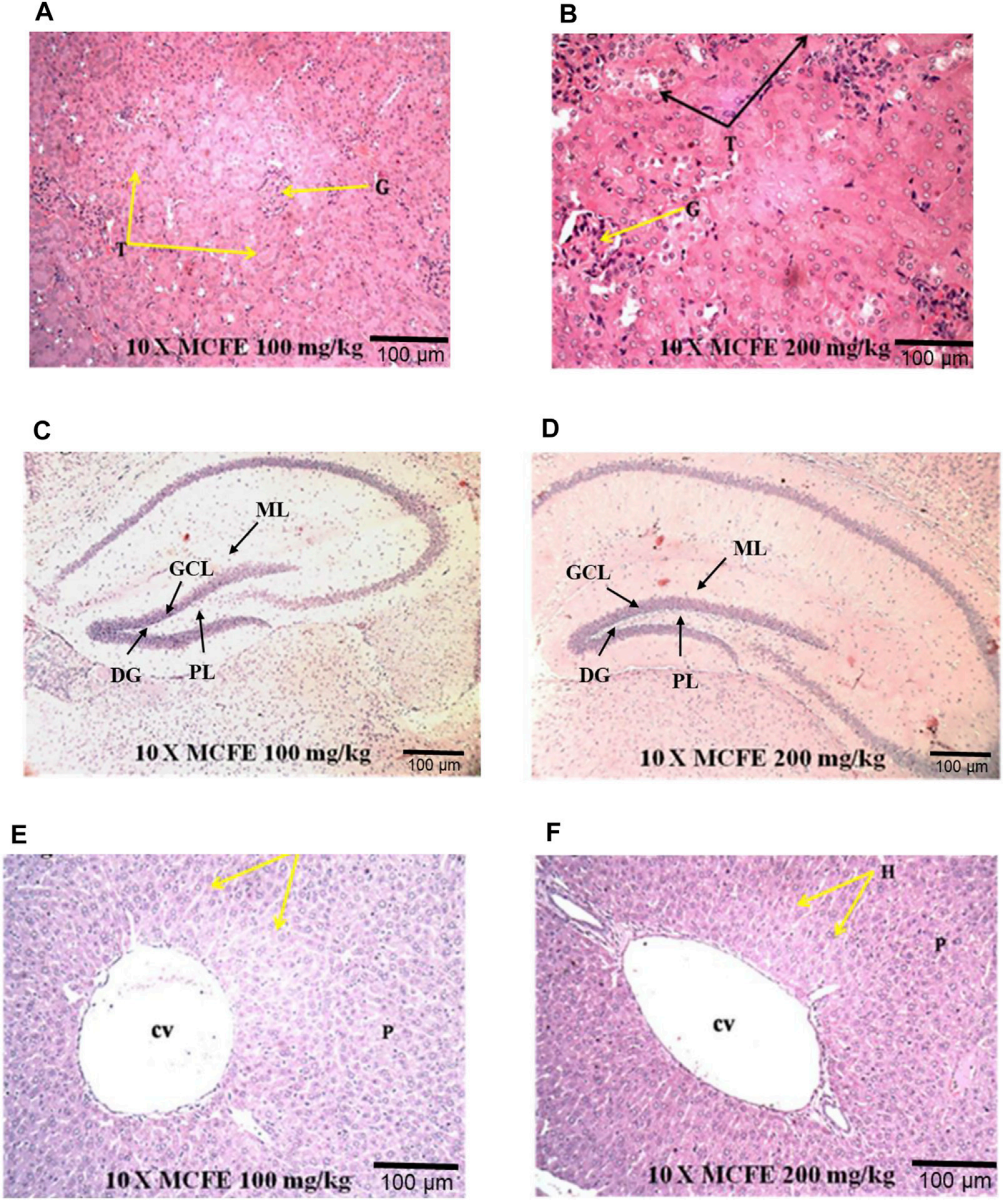
Photomicrographs at 10× magnification of haematoxylin and eosin (H&E) stained kidney **(A)** and **(B)**; brain **(C)** and **(D)**; liver **(E)** and **(F)**, when MCFE is administered at different doses in mice model. *(G) Glomerulus, (T) Tubules; (DG) Dentate gyrus, (GCL) Granule cell layer, (ML) Molecular layer, (PL) Polymorphic layer; Central vein (cv), Parenchyma (p).

In the present study, the histological examination of the kidney sections of both MCFE (100 and 200 mg/kg)-treated groups showed normal architecture of renal corpuscle and tubules, proximal convoluted tubules and distal convoluted tubules [Figures 6A,B]. Brain histology examination of the MCFE-treated group at both 100 and 200 mg/kg showed a normal histological architecture. The pia matter, molecular, granular and pyramidal layers appeared normal with no signs of necrosis with no hemorrhagic symptoms [Figures 6C,D]. The histopathological examination of the liver sections in the MCFE at both doses was also normal where the central vein lies at the centre of the lobule surrounded by the hepatocytes with strongly eosinophilic granulated cytoplasm and distinct nuclei [Figure 6E,F].

**FIGURE 7:**
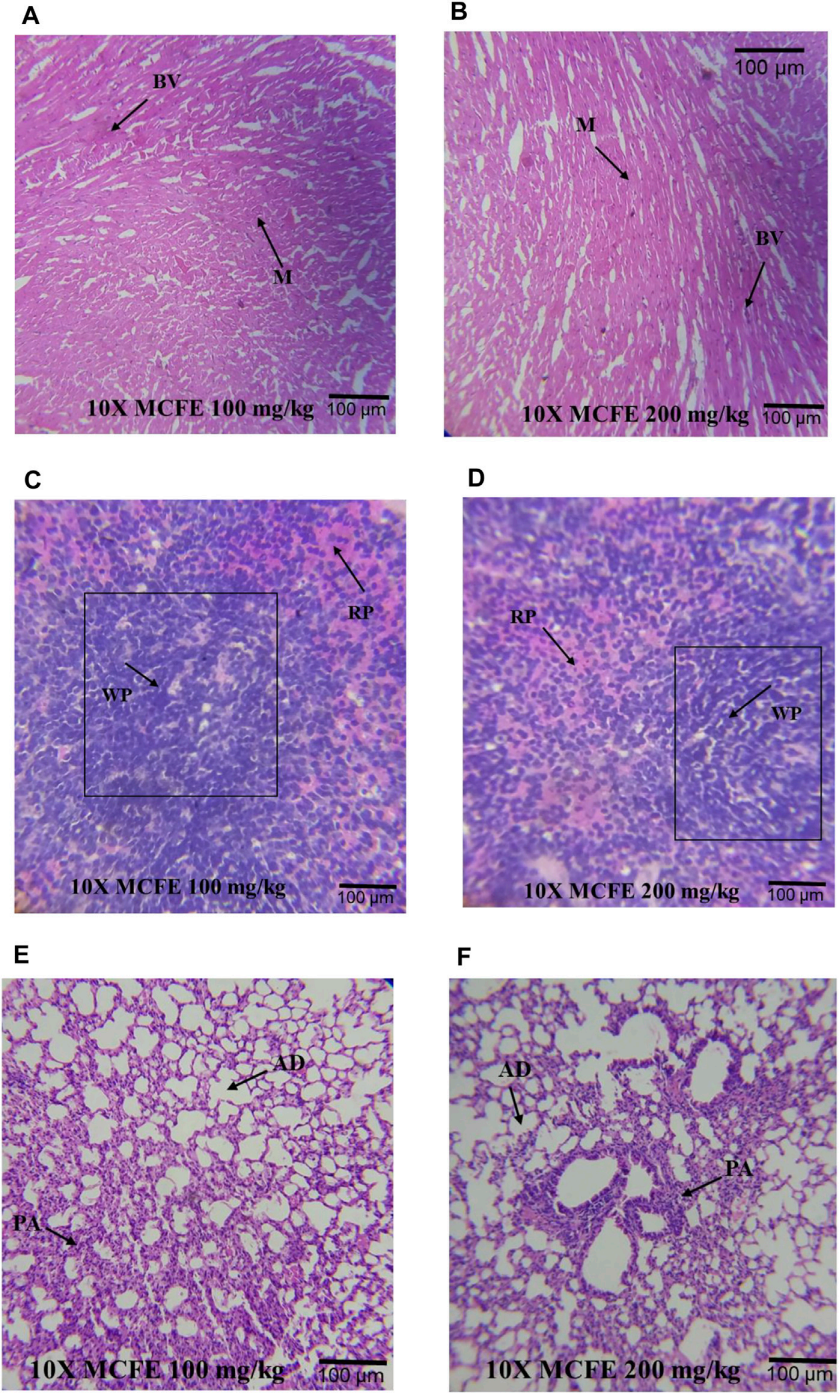
Photomicrographs at 10× magnifications of haematoxylin and eosin (H&E) stained heart **(A)** and **(B)**; spleen **(C)** and **(D)**; lung **(E)** and **(F)**, when MCFE is administered at different doses. *Blood vessels (BV), Myocardium (M), Red pulp (red) (RP), White pulp (WP), Pulmonary artery (PA) and Alveolar duct (AD).

Similarly, other organs were normal [the heart: Figures 7A,B, the spleen: Figures 7C,D as well as for the lungs: Figures 7E,F].

## 4 Discussion

**FIGURE 8:**
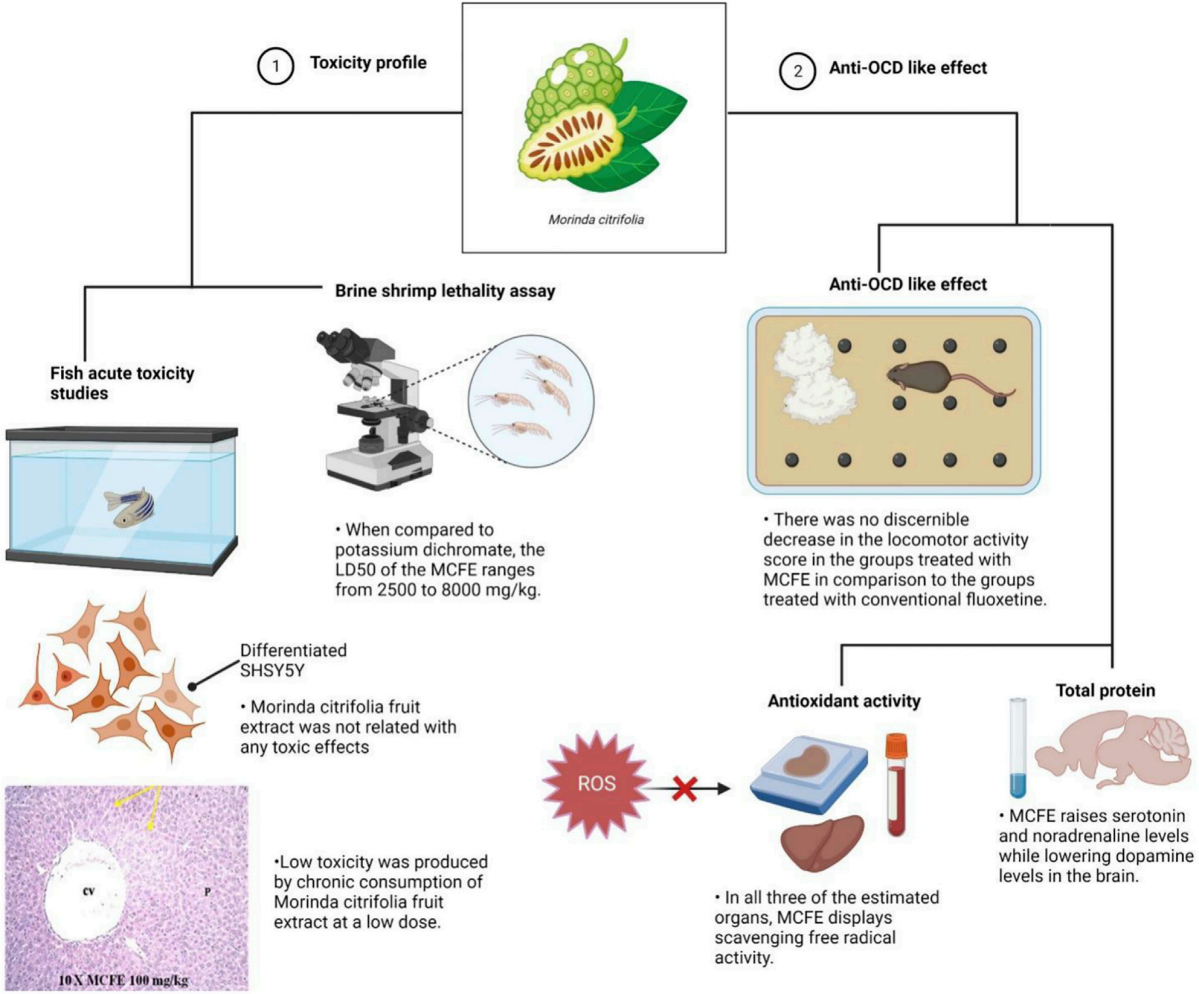
Schematic illustration depicted MCFE toxicity profile and anti-OCD-like effects. Aside from its significant neuroprotective properties, the results of the experiments conducted revealed a minimal toxicity profile of the extract in both *in vitro* and *in vivo* models.

This is the first comprehensive study to report on the toxicity profile and the anti-OCD like effect of MCFE (Figure 8). The LD_50_ of was determined to be between 2,500—8,000 mg/kg. In the interest of animal welfare and efficient use of resources, it is important to avoid the unnecessary use of animals whenever possible. In the field of aquatic toxicology, this fact especially applies to the acute toxicity testing of fish according to the OECD Test Guideline 203. The threshold approach described in the guideline addresses fish toxicity by initially using a single concentration test (limit test) as the threshold concentration (TC), thus requiring fewer fish, as compared to conducting the full acute fish toxicity study. No toxicity effects were associated with MCFE at the concentration tested in the fish acute toxicity test according to OECD TG 203 (limit test) with an LC_50_ of >100 mg/L suggesting that no further testing is required as per the guideline.

The acute toxicity data obtained from our study provide a baseline information on the concentration of MCFE to be used for further investigations which requires investigation of the chronic effects, such as fish growth, survival rate and reproduction. Furthermore, the evaluation of MCFE on cell viability in differentiated SHSY5Y cells in the presence of glutamate (20 mM) was conducted confirmed the reduction in the percentage cell viability of glutamate-treated cells against the combined treatment with glutamate and MCFE at 100, 200, 400, and 800 μg/ml.

In the marble burying test, the total activity score was recorded for all treatment groups for 10 min after evaluation of marble burying behavior test that was conducted for 30 min. There was a significant decrease in the number of beam interactions (locomotor count) in the negative control (diazepam-treated) as compared to the standard group (fluoxetine-treated) where there were no significant decrease in the beam interactions (locomotor count) indicating that animal locomotion was not affected. Additionally, there was no significant reduction in the locomotor activity score in the MCFE-treated groups as compared to the standard fluoxetine-treated group, indicating that locomotion was unaffected by fluoxetine and MCFE treatments. Biswas et al. (2021), used marble burying test and has taken *Psidium guajava* L. extract which shows non-significant decrease at 200 mg/kg BW but at 400 mg/kg BW shows a statistically significant decrease in the number of marble buried compared with control group which shows dose dependent manner and also suggested marble burying test as beneficial model for evaluating drugs for anti-obsessive-compulsive disorder. *M. citrifolia* in marble burying test indicates that increases of dose of extract, significantly decrease the number of marble buried. Similarly, based on the nestlet shredding test, it is evident that the % nestlet weight reduction before and after exposure to the animal was related to the anti-compulsive effect. Furthermore, the % nestlet shredded activity of MCFE was in a dose-dependent manner. Chang et al. (2014) suggested that the length of time before shredding starts is a reflection of the animals’ levels of exploratory activity and their drive to engage in natural behavior. Therefore, these elements in addition to repetitious behavior may have covered up a possible impact of the Hfe mutation on both nestlet-shredding score and the time delay before the start of shredding and findings showed that the latency rose in dose dependent manner.

Total protein measurement confirmed that there was a significant decrease in the total protein in the diazepam-treated groups as compared to the control group. Additionally, the amount of DA was reduced in diazepam-treated groups while NA and 5-HT levels were increased in fluoxetine and MCFE groups when compared to the control. These results are comparable to a preliminary study by Morgese et al. (2022), suggesting the involvement of both 5-HT and DA systems in OCD. Obsessive-compulsive symptoms may result from an imbalance in activity between 5-HT and DA function in relevant brain regions. The imbalance may stem from an absolute change in DA function, 5-HT function, or both (Festucci et al., 2022).

A study provided evidence for imbalanced mono-aminergic neurotransmitter modulation in OCD. The success of pharmacological treatment of OCD with serotonin reuptake inhibitors and atypical antipsychotic drugs suggests that both the central serotonergic and dopaminergic systems are involved in the pathophysiology of the disorder (Koepsell, 2021). It is evident form the present investigation that MCFE decreases the brain DA levels while increasing 5-HT and NA levels which supports the involvement of monoamines in the treatment of OCD and MCFE.

In OCD, oxidative processes occur and are associated with inflammation and dysfunction in neurotransmitters. There is an overall oxidative imbalance occurring due to either a rebound phenomenon or chronicity of the condition. Antidepressants used in the treatment of OCD may offer some antioxidant effects (Maia et al., 2019). Various studies have reported elevated levels of MDA, SOD, glutathione peroxidase and CAT; considered as oxidative stress markers (Kar and Choudhury, 2016) in patients with OCD. Hence, in this study, we evaluated MCFE for its antioxidant effects on various tissues like the brain, liver and kidney, to correlate it with OCD. There was a slight increase in the antioxidant levels of fluoxetine and MCFE-treated groups although the increment was not statistically significant, thus suggesting that MCFE exhibits neuroprotection by scavenging free radical activity in all three organs.

Following MCFE administration, the histopathology of the brain, liver and kidney tissues showed normal morphological structure with no toxicity and no indication of deformities. These reports were in accordance to a previous study by Hadijah et al. (2003) who conducted the acute and sub-chronic toxicity studies of an aqueous extract of *M. citrifolia* fruit in rats. In the LD_50_ test, rats were given three dosages of *M. citrifolia* extracts (1.0, 2.0 and 3.0 g/kg). They were observed for any toxic signs, especially death in the first 24 h (and continued up to 14 days). In the sub-chronic study, the effects of *M. citrifolia* extracts in rats (0.25, 0.50, and 1.0 g/kg) were determined for 6 weeks by measuring the blood biochemical parameters. The study reported no signs of toxicity (Hadijah et al., 2003) as were confirmed in our study. In another study, the chronic toxicity evaluation of *M. citrifolia* fruit and leaf in mice was performed by Shalan et al. (2017) It was concluded that chronic intake of high dose *Morinda* fruit extract (2 mg/ml) damaged the mice’s liver. Chronic intake of low dose MCFE (1 mg/ml) however, produced a low toxicity. Chronic intake of *M. citrifolia* leaf extract produced no toxicity. However, this article received strong criticism from West 2017 (West, 2017) who suggested that there are differences between the commercial Noni preparations from the extracts prepared with additional requirements (West, 2017).

Limitation of study to perform *in vivo* evaluation of *M. citrifolia* on pharmacological induced animal models of OCD and to identify the pathway responsible for the activity and also to perform safety and efficacy studies of *M. citrifolia* at preclinical and clinical stages.

**FIGURE 9:**
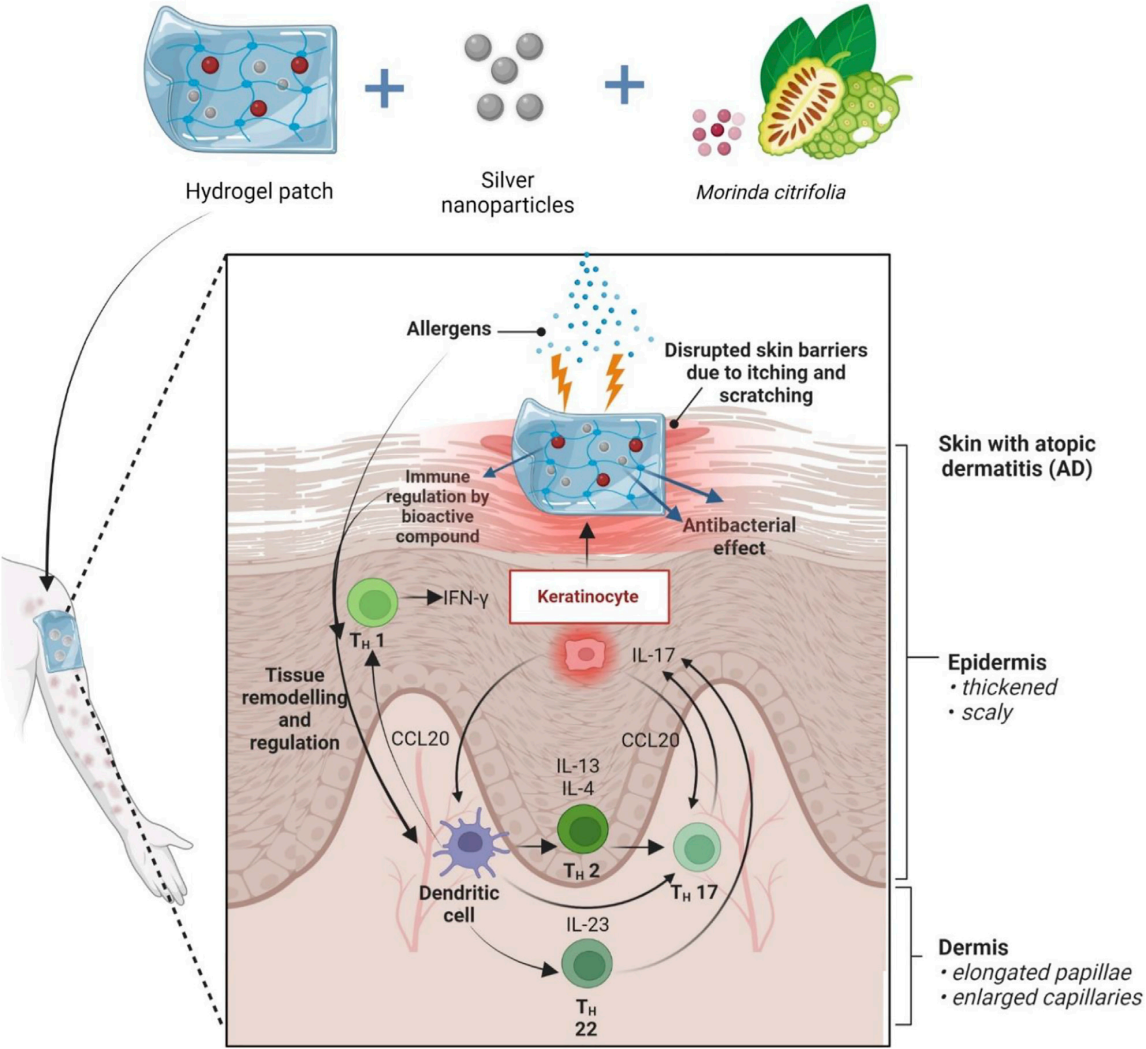
Future prospect of MCFE incorporated in hydrogel patch embedded silver nanoparticles. Allergens cause itching, which weakens the skin’s defenses. The fruit extract is thought to be able to regulate the expression of Th1, Th2, Th17, and Th22-mediated cytokines including Interleukin (IL)-4, IL-5, IL-13, and IL-31 in skin lesions that induce aggravation of associated symptoms with atopic dermatitis. Higher therapeutic efficacy is expected when combined with nanocarriers.

Apart from its use in psychiatric-related disorders, *M. citrifolia* fruit extract has also been shown to be able to modulate the immune response in atopic dermatitis, suggesting that it may be used as a therapeutic agent to lessen skin lesions (Kim et al., 2020). Long-lasting (chronic) atopic dermatitis can be triggered by a variety of things, including psychological stress (Suárez et al., 2012), environmental factors (Alexander et al., 2020), as well as genetics (Løset et al., 2019), and it tends to flare up often. Disruption of the skin’s protective barriers brought on by persistent scratching as a result of the irritation it causes will make it more susceptible to bacterial infections, such as the skin-colonizing *Staphylococcus aureus* (Alexander et al., 2020). Despite the effective utilisation of plant extracts, natural products often pose difficulties for drug development, such as technical obstacles to screening, isolation, characterization, and optimization, which led to a drop in their development and exploration in the industry (Løset et al., 2019). In the years to come, we anticipate greater progress may be made in the development of strategies to get through these constraints. As for the future perspectives, this paper recommends loading the fruit extract within a nanocarrier such as hydrogel embedded with metal nanoparticles to be used in the management of AD. By including the extract inside the vehicle, the active components will be delivered in a more targeted and localised manner, improving the extract’s bioavailability, dose, and capacity for skin permeation and retention (Atanasov et al., 2021). Transepidermal Water Loss (TEWL) was also not significantly affected by the choice of hydrogel delivery vehicle when compared to other formulations (Kircik, 2012; Harrison and Spada, 2018). Previous research has shown that electrospun polymeric nanofibrous scaffold containing bioactive components derived from *M. citrifolia* served as an effective cutaneous wound healing therapy by stimulating wound healing proteins glycogen-synthase kinase-3-*β*-protein and promoting the proliferation and adhesion of human skin keratinocytes (Ekambaram et al., 2021). Possessing antibacterial properties, metal nanoparticles such as silver nanoparticles have the potential to eliminate additional bacterial infection threats (Keck and Schwabe, 2009; Damiani et al., 2019; Mussin et al., 2021; Permana et al., 2021) and thus may be beneficial as used together in the management of skin lesions (Figure 9).

## 5 Conclusion

The marble burying and nestlet shredding tests offer precise and sensitive evaluations of repetitive and compulsive-like behavior in rats. These tests would also be helpful in the evaluation of repetitive behaviours following traumatic brain injury and animal exposure to chronic stress because each of these conditions can lead to compulsive-like behaviours in both humans and animals. This is the first study to demonstrate that MCFE dose-dependently attenuated the behaviours marble burying and nestlet shredding behaviors in mice, and the effect was comparable to that of the reference standard drug, fluoxetine. According to the results of the current investigation, exhibits anti-compulsive effects that are dose dependent. In this study, we demonstrated that MCFE treatment for OCD similar to selective serotonin reuptake inhibitors (SSRIs) as compared to the diazepam treated group when used at a comparative lower dose. Further, this study revealed that *M. citrifolia’s* antidepressant to sedative-like effects shifted in a dose-dependent manner. The antioxidants present in the MCFE may account for the ability to inhibit the marble burying behaviour without affecting the locomotion of the animal, as well for nestlet shredding test. It may be concluded that the MCFE produces an anti OCD like activity that seems to be dependent on its interaction with the serotonergic, noradrenergic and dopaminergic systems as evident from the brain monoamine measurement of MCFE produces an anti-OCD like activity that seems to be dependent on its interaction with the serotonergic, noradrenergic and dopaminergic systems as evident from the brain monoamine measurement. Despite the study limitations, the present research able to demonstrate that MCFE alleviates OCD by restoring dopamine and serotonin levels to normal and reducing inflammation in the brain. Further to perform *in vivo* evaluation of *M. citrifolia* on pharmacological induced animal models of OCD and to identify the pathway responsible for the activity and also to perform safety and efficacy studies of *M. citrifolia* at preclinical and clinical stages.

## Data Availability

The raw data supporting the conclusions of this article will be made available by the authors, without undue reservation.

## References

[B1] Agrawal A. Gopal K. (2013). “General principles of toxicity and its application,” in Biomonitoring of water and waste water. New Delhi: Springer, 101–108.

[B2] Ahmadzadeh M. Esmaeilzadeh Z. Khezri M. R. Jafari A. Ghasemnejad-Berenji M. (2022). The promising effect of topiramate on random-pattern skin flap survival in rats. Aesthetic Plast. Surg., 1–8. 10.1007/s00266-022-02969-6 35715535

[B3] Ajibade M. A. Akhigbemen A. M. Okolie N. P. Ozolua R. I. (2022). Methanol leaf extract of Paullinia pinnata exerts sleep-enhancing and anticonvulsant effects via a mechanism involving the GABAergic pathway. Epilepsy Res. 183, 106943. 10.1016/j.eplepsyres.2022.106943 35636276

[B4] Alexander H. Paller A. S. Traidl-Hoffmann C. Beck L. A. De Benedetto A. Dhar S. (2020). The role of bacterial skin infections in atopic dermatitis: Expert statement and review from the international eczema Council skin infection group. Br. J. Dermatol. 182 (6), 1331–1342. 10.1111/bjd.18643 31677162 PMC7317931

[B5] Ali M. Ansari S. H. Ali S. Khan S. A. Alam S. Hussain A. (2013). Phytochemical and pharmacological potential updates of *Morinda citrifolia* linn.(noni). Res. J. Pharm. Technol. 6 (11), 1285–1294. 10.5958/0974-360X

[B6] Atanasov A. G. Zotchev S. B. Dirsch V. M. Orhan I. E. Banach M. Rollinger J. M. (2021). Natural products in drug discovery: Advances and opportunities. Nat. Rev. Drug Discov. 20 (3), 200–216. 10.1038/s41573-020-00114-z 33510482 PMC7841765

[B7] Bauer B. Mally A. Liedtke D. (2021). Zebrafish embryos and larvae as alternative animal models for toxicity testing. Int. J. Mol. Sci. 22 (24), 13417. 10.3390/ijms222413417 34948215 PMC8707050

[B8] Biswas S. Mondol D. Jodder P. Sana S. Saleh M. Tarafdar A. K. (2021). Evaluation of neurobehavioral activities of ethanolic extract of Psidium guajava Linn leaves in mice model. Futur. J. Pharm. Sci. 7 (1), 36–42. 10.1186/s43094-021-00188-5

[B9] Bose B. Tripathy D. Chatterjee A. Tandon P. Kumaria S. (2019). Secondary metabolite profiling, cytotoxicity, anti-inflammatory potential and *in vitro* inhibitory activities of Nardostachys jatamansi on key enzymes linked to hyperglycemia, hypertension and cognitive disorders. Phytomedicine 55, 58–69. 10.1016/j.phymed.2018.08.010 30668444

[B10] Bottemanne H. Arnould A. (2021). Ketamine augmentation of exposure response prevention therapy for obsessive-compulsive disorder. Innov. Clin. Neurosci. 18 (10-12), 9–11. PMC8794478 35096475

[B11] Chang J. Kueon C. Kim J. (2014). Influence of lead on repetitive behavior and dopamine metabolism in a mouse model of iron overload. Toxicol. Res. 30 (4), 267–276. 10.5487/TR.2014.30.4.267 25584146 PMC4289927

[B12] Damiani G. Eggenhöffner R. Pigatto P. D. M. Bragazzi N. L. (2019). Nanotechnology meets atopic dermatitis: Current solutions, challenges and future prospects. Insights and implications from a systematic review of the literature. Bioact. Mater 4, 380–386. 10.1016/j.bioactmat.2019.11.003 31872162 PMC6909150

[B13] Deutch C. E. (2018). Browning in apples: Exploring the biochemical basis of an easily-observable phenotype. Biochem. Mol. Biol. Educ. 46 (1), 76–82. 10.1002/bmb.21083 28843018

[B14] Ding X. Han C. Hu W. Fu C. Zhou Y. Wang Z. (2022). Acute and subacute safety evaluation of black tea extract (herbt tea essences) in mice. Toxics 10 (6), 286. 10.3390/toxics10060286 35736895 PMC9228953

[B15] Ekambaram R. Sugumar M. Swaminathan E. Micheal Raj A. P. Dharmalingam S. (2021). Design and fabrication of electrospunMorinda citrifolia-based nanofibrous scaffold as skin wound dressing material:in vitroandin silicoanalysis. Biomed. Mat. 16, 045014. 10.1088/1748-605X/abef59 33725680

[B16] Festucci F. Annunzi E. Pepe M. Curcio G. D'Addario C. Adriani W. (2022). Dopamine-transporter heterozygous rats carrying maternal wild-type allele are more vulnerable to the development of compulsive behavior. Synapse 76 (9–10), 31–44. 10.1002/syn.22244 35772468

[B17] Foltran R. B. Stefani K. M. Höcht C. Diaz S. L. (2020). Neurochemical, behavioral, and neurogenic validation of a hyposerotonergic animal model by voluntary oral consumption of para-chlorophenylalanine. ACS Chem. Neurosci. 11 (6), 952–959. 10.1021/acschemneuro.9b00687 32107912

[B18] Goodwin G. M. (2022). The overlap between anxiety, depression and obsessive-compulsive disorder. Dialogues Clin. Neurosci. 17, 249–260. 10.31887/DCNS.2015.17.3/ggoodwin PMC4610610 26487806

[B19] Hadijah H. Ayub M. Y. Zaridah H. Normah A. (2003). Acute and subchronic toxicity studies of an aqueous extract of *Morinda citrifolia* fruit in rats. J. Trop. Agric. Food Sci. 31, 67–74.

[B20] Harrison I. P. Spada F. (2018). Hydrogels for atopic dermatitis and wound management: A superior drug delivery vehicle. Pharmaceutics 10, E71. 10.3390/pharmaceutics10020071 29899219 PMC6027388

[B21] Kar S. K. Choudhury I. (2016). An empirical review on oxidative stress markers and their relevance in obsessive-compulsive disorder. Int. J. Nutr. Pharmacol. Neurol. Dis. 6 (4), 139. 10.4103/2231-0738.191641

[B22] Karpinski M. Mattina G. F. Steiner M. (2017). Effect of gonadal hormones on neurotransmitters implicated in the pathophysiology of obsessive-compulsive disorder: A critical review. Neuroendocrinology 105 (1), 1–16. 10.1159/000453664 27894107

[B23] Keck C. M. Schwabe K. (2009). Silver-nanolipid complex for application to atopic dermatitis skin: Rheological characterization, *in vivo* efficiency and theory of action. J. Biomed. Nanotechnol. 5 (4), 428–436. 10.1166/jbn.2009.1053 20055090

[B24] Kim S. H. Seong G. S. Choung S. Y. (2020). Fermented *Morinda citrifolia* (noni) alleviates DNCB-induced atopic dermatitis in NC/nga mice through modulating immune balance and skin barrier function. Nutrients 12 (1), E249. 10.3390/nu12010249 31963703 PMC7019744

[B25] Kircik L. H. (2012). Transepidermal water loss (TEWL) and corneometry with hydrogel vehicle in the treatment of atopic dermatitis: A randomized, investigator-blind pilot study. J. Drugs Dermatol. 11, 180–184. 22270199

[B26] Koepsell H. (2021). General overview of organic cation transporters in brain. Handb. Exp. Pharmacol. 266, 1–39. 10.1007/164_2021_449 33782773

[B27] Lairion F. Saporito-Magriñá C. Musacco-Sebio R. Fuda J. Torti H. Repetto M. G. (2022). Nitric oxide, chronic iron and copper overloads and regulation of redox homeostasis in rat liver. J. Biol. Inorg. Chem. 27 (1), 23–36. 10.1007/s00775-021-01908-1 34791544

[B28] Løset M. Brown S. J. Saunes M. Hveem K. (2019). Genetics of atopic dermatitis: From DNA sequence to clinical relevance. Dermatology 235 (5), 355–364. 10.1159/000500402 31203284

[B29] Magi S. Piccirillo S. Maiolino M. Lariccia V. Amoroso S. (2020). NCX1 and EAAC1 transporters are involved in the protective action of glutamate in an *in vitro* Alzheimer's disease-like model. Cell Calcium 91, 102268. 10.1016/j.ceca.2020.102268 32827867

[B30] Maia A. Oliveira J. Lajnef M. Oliva-Maia A. J. Tamouza R. LeboyerM. (2019). Oxidative and nitrosative stress markers in obsessive-compulsive disorder: A systematic review and meta-analysis. Acta Psychiatr. Scand. 139 (5), 420–433. 10.1111/acps.13026 30873609

[B31] Malta C. P. Silva Barcelos R. C. Segat H. J. Burger M. E. Souza Bier C. A. Morgental R. D. (2022). Toxicity of bioceramic and resinous endodontic sealers using an alternative animal model: *Artemia salina* . J. Conserv. Dent. 25 (2), 185–188. 10.4103/jcd.jcd_401_21 35720815 PMC9205353

[B32] Morgese M. G. Bove M. Di Cesare Mannelli L. Schiavone S. Colia A. L. Dimonte S. (2022). Precision medicine in alzheimer's disease: Investigating comorbid common biological substrates in the rat model of amyloid beta-induced toxicity. Front. Pharmacol. 12, 799561. 10.3389/fphar.2021.799561 35046821 PMC8763383

[B33] Murphy M. Mills S. Winstone J. Leishman E. Wager-Miller J. Bradshaw H. (2017). Chronic adolescent d^9^-tetrahydrocannabinol treatment of male mice leads to long-term cognitive and behavioral dysfunction, which are prevented by concurrent cannabidiol treatment. Cannabis Cannabinoid Res. 2 (1), 235–246. 10.1089/can.2017.0034 29098186 PMC5655843

[B34] Mushtaq S. Abbasi B. H. Uzair B. Abbasi R. (2018). Natural products as reservoirs of novel therapeutic agents. EXCLI J. 17, 420–451. 10.17179/excli2018-1174 29805348 PMC5962900

[B35] Mussin J. Robles-Botero V. Casañas-Pimentel R. Rojas F. Angiolella L. San Martín-Martínez E. (2021). Antimicrobial and cytotoxic activity of green synthesis silver nanoparticles targeting skin and soft tissue infectious agents. Sci. Rep. 11 (1), 14566. 10.1038/s41598-021-94012-y 34267298 PMC8282796

[B36] Ohkawa H. Onishi N. Yagi K. (1979). Assay for lipid peroxides in animal tissues by thiobarbituric acid reaction. Anal. Biochem. 95, 351–358. 10.1016/0003-2697(79)90738-3 36810

[B37] Permana A. D. Anjani Q. K. Sartini U. E. Volpe-Zanutto F. Paredes A. J. Evary Y. M. (2021). Selective delivery of silver nanoparticles for improved treatment of biofilm skin infection using bacteria-responsive microparticles loaded into dissolving microneedles. Mat. Sci. Eng. C Mat. Biol. Appl. 120, 111786. 10.1016/j.msec.2020.111786 33545912

[B38] Samarghandian S. Azimi-Nezhad M. Farkhondeh T. Samini F. (2017). Anti-oxidative effects of curcumin on immobilization-induced oxidative stress in rat brain, liver and kidney. Biomed. Pharmacother. 87, 223–229. 10.1016/j.biopha.2016.12.105 28061405

[B39] Shahini N. Talaei A. Shalbafan M. Faridhosseini F. Ziaee M. (2021). Effects of celecoxib adjunct to selective serotonin reuptake inhibitors on obsessive-compulsive disorder. Basic Clin. Neurosci. 12 (4), 489–498. 10.32598/bcn.2021.1998.1 35154589 PMC8817183

[B40] Shalan N. A. Mustapha N. M. Mohamed S. (2017). Chronic toxicity evaluation of *Morinda citrifolia* fruit and leaf in mice. Regul. Toxicol. Pharmacol. 83, 46–53. 10.1016/j.yrtph.2016.11.022 27871867

[B41] Solomon N. (1999a). Noni juice (*Morinda citrifolia*): The tropical fruit with 101 medicinal uses. 2nd Ed. Utah, United States: Woodland Publishing.

[B42] Solomon N. (1999b). The noni Phenomenon. 2nd Ed. Utah, United States: Direct Source Publishing.

[B43] Suárez A. L. Feramisco J. D. Koo J. Steinhoff M. (2012). Psychoneuroimmunology of psychological stress and atopic dermatitis: Pathophysiologic and therapeutic updates. Acta Derm. Venereol. 92 (1), 7–15. 10.2340/00015555-1188 22101513 PMC3704139

[B44] West B. J. (2017). Report of *Morinda citrifolia* chronic toxicity not applicable to commercial noni juice. Regul. Toxicol. Pharmacol. 88, 360–361. 10.1016/j.yrtph.2017.03.014 28341591

[B45] Wulaer B. Nagai T. Sobue A. Kuroda K. Kaibuchi K. Yamada K. (2018). Repetitive and compulsive-like behaviors lead to cognitive dysfunction in Disc1^Δ2-3/Δ2-3^ mice. Genes Brain Behav. 17 (8), e12478. 10.1111/gbb.12478 29635888

[B46] Zheng Y. Xiao L. Xie Y. Wang H. Wang G. (2020). Prevalence and characteristics of obsessive-compulsive disorder among urban residents in Wuhan during the stage of regular control of coronavirus disease-19 epidemic. Front. Psychiatry 11, 594167. 10.3389/fpsyt.2020.594167 33391055 PMC7772465

